# Structure, Function, and Regulation of the Essential Virulence Factor Capsular Polysaccharide of *Vibrio vulnificus*

**DOI:** 10.3390/ijms21093259

**Published:** 2020-05-05

**Authors:** Gregg S. Pettis, Aheli S. Mukerji

**Affiliations:** Department of Biological Sciences, Louisiana State University, Baton Rouge, LA 70803, USA; amuker1@lsu.edu

**Keywords:** *Vibrio*, capsular polysaccharide, phase variation, sepsis, wound infection

## Abstract

*Vibrio vulnificus* populates coastal waters around the world, where it exists freely or becomes concentrated in filter feeding mollusks. It also causes rapid and life-threatening sepsis and wound infections in humans. Of its many virulence factors, it is the *V. vulnificus* capsule, composed of capsular polysaccharide (CPS), that plays a critical role in evasion of the host innate immune system by conferring antiphagocytic ability and resistance to complement-mediated killing. CPS may also provoke a portion of the host inflammatory cytokine response to this bacterium. CPS production is biochemically and genetically diverse among strains of *V. vulnificus*, and the carbohydrate diversity of CPS is likely affected by horizontal gene transfer events that result in new combinations of biosynthetic genes. Phase variation between virulent encapsulated opaque colonial variants and attenuated translucent colonial variants, which have little or no CPS, is a common phenotype among strains of this species. One mechanism for generating acapsular variants likely involves homologous recombination between repeat sequences flanking the *wzb* phosphatase gene within the Group 1 CPS biosynthetic and transport operon. A considerable number of environmental, genetic, and regulatory factors have now been identified that affect CPS gene expression and CPS production in this pathogen.

## 1. Introduction

*Vibrio vulnificus* is a natural inhabitant of estuarine and other coastal marine environments where it exists free living or becomes concentrated in filter-feeding molluscan shellfish, such as oysters. The bacterium gains entrance to the human host through consumption of contaminated water or food, particularly raw or undercooked oysters and other seafood. Alternatively, infection occurs through open wounds that contact seawater or shellfish containing the bacterium [1]. *V. vulnificus* notoriously causes an acute fulminating septicemia as well as severe wound infections. A third illness, gastroenteritis, is not life threatening and is typically not reported. Septicemic patients succumb to death in as little as 24 hours and in over 50% of cases. *V. vulnificus* is responsible for the vast majority of reported deaths associated with seafood consumption in the U.S. [1]. Patients developing sepsis through oral ingestion of the bacterium overwhelmingly have an underlying predisposing health condition such as liver disease, AIDS, diabetes, hemachromatosis, cancer, or a compromised immune system [2]. Infection of wounds occurs typically during swimming, fishing, or handling of seafood. Such infections can progress rapidly to a necrotizing fasciitis at the site of infection, and they often require debridement or amputation of the affected limb in order to prevent death, which occurs in ~25% of cases [3,4]. 

A number of factors contribute to virulence of *V. vulnificus*, including its acid resistance, as well as its production of capsular polysaccharide (CPS), lipopolysaccharide (LPS), iron acquisition systems, cytotoxic factors, and motility and adherence/adhesion molecules [3]. Unlike most of the other contributing factors, however, CPS is considered to be essential for virulence, since it confers the ability to circumvent the host immune response [3,5]. Here, we summarized research aimed at elucidating *V. vulnificus* CPS structure, function, and genetics, as well as control of CPS production via phase variation and other known environmental, genetic, and regulatory factors. 

## 2. Identification of the *V. vulnificus* Capsule and Its Correlation with Virulence 

Kreger et al. (1981) first presented evidence that virulence of *V. vulnificus* was due, at least in part, to an antiphagocytic surface molecule [6]. In a follow-up study, physicochemical analysis of the partially purified molecule suggested that it was a heat-sensitive acidic polysaccharide [7]. Ruthenium red staining, coupled with transmission electron microscopy, provided evidence that this polysaccharide densely coated the cell surface of a virulent strain while it was present in reduced amounts on the surface of a weakly virulent one [7]. Amako et al. (1984) showed the ruthenium red staining patterns of two *V. vulnificus* strains, namely FCC and ATCC 27562 (Table 1), were distinct: while both strains showed a mixed population of stained and unstained cells, the proportion of stained cells was greater for strain FCC than ATCC 27562. Moreover, the increased proportion of stained cells correlated with increased virulence in mice and to resistance to the bacteriocidal action of normal human serum (NHS). These authors referred to this surface polysaccharide as a capsule [8]. 

**Figure 1:**
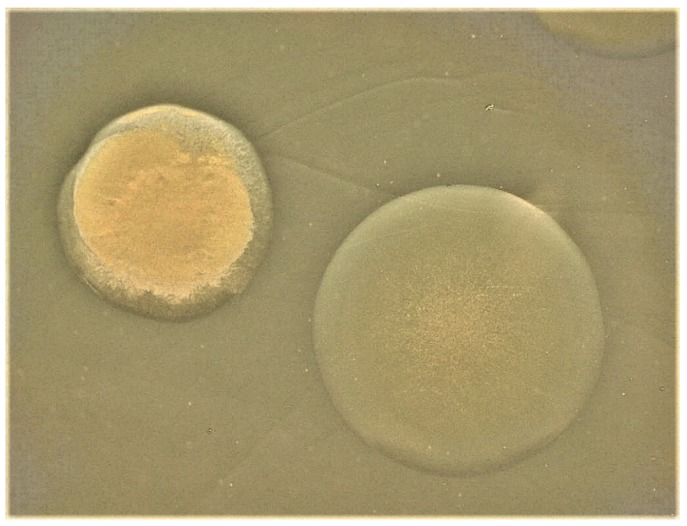
Opaque (left) and translucent (right) colony types of *V. vulnificus*.

Separately, variation in capsule production among four other *V. vulnificus* strains was similarly characterized [9]. Opaque colony types of strains consisted of encapsulated cells while the cells of translucent colony types produced little or no observable capsule (see Figure 1 for examples of opaque and translucent colony types of *V. vulnificus*). The presence of capsular material again correlated with increased virulence in mice and with resistance to NHS, as well as with antiphagocytic ability, and increased invasiveness in subcutaneous tissue of guinea pigs [9]. In a more comprehensive study, Simpson et al. (1987) examined a larger number of both clinical and environmental isolates for colony phenotype, virulence in mice, and other properties. Twenty-six virulent strains produced both opaque and translucent colony types, while ten strains displayed only the translucent colony type and were avirulent [10]. Thus, a correlation between CPS and virulence in *V. vulnificus* was apparent. 

This relationship was subsequently explored further using transposon mutagenesis. Compared to the opaque clinical isolate MO6-24/O (Table 1), two translucent transposon mutant derivatives appeared acapsular in carbohydrate staining procedures and were attenuated by several orders of magnitude for virulence in mice [11]. Moreover, while only opaque colony-yielding bacteria were recovered post infection from surviving mice infected with MO6-24/O, only bacteria of a translucent colony type (or no bacteria at all) were recovered from mice infected with the acapsular transposon mutants. A spontaneous translucent variant of MO6-24/O, strain MO6-24/T (Table 1), which showed reduced CPS on its cell surface, was also attenuated for virulence though not to the same magnitude as the acapsular transposon mutants. Bacteria recovered from mice infected with MO6-24/T all had an opaque morphology, indicating that phase switching from translucent back to opaque had likely occurred in vivo by an undetermined mechanism [11]. Attenuation of virulence was a common property seen for acapsular transposon mutants of *V. vulnificus* in other studies as well [12,13,14].

## 3. CPS Composition and Structure 

Carbohydrate composition of *V. vulnificus* CPS was first reported for the clinical isolate MO6-24/O following extensive purification of its capsular material and analysis by nuclear magnetic resonance (NMR) spectroscopy. The MO6-24/O CPS polymer consists of four-sugar repeat subunits, each containing three residues of 2-acetamido-2,6-dideoxyhexopyranose in the α-gluco configuration (trivially known as *N*-acetyl quinovosamine (QuiNAc)) and one residue of 2-acetamido hexuronate in the α-galactopyranose configuration (*N*-acetyl-galactosamine uronic acid (GalNAcA)) [15]. Further structural details, including stereochemistry of sugar residues and position of glycosidic linkages, were provided by heteronuclear NMR spectroscopy and high-performance anion-exchange chromatography (HPAEC). In the same study, CPS with the identical composition was detected for the spontaneous translucent variant MO6-24/T, but not for an acapsular transposon mutant derivative [15]. Similar analysis of the CPS produced by another clinical isolate (BO62316) (Table 1) also revealed evidence of a four-sugar repeating subunits that included QuiNAc and GalNAcA residues but also included other sugars that were distinct from MO6-24/O CPS [16]. Analysis of the CPS from clinical isolate ATCC27562 showed further diversity with respect to composition, including the presence of *N*-acetyl muramic acid, a well-known component of peptidoglycan, as well as evidence that the four-sugar repeat subunits for this strain are linked to serine via an amide bond [17]. 

Purified *V. vulnificus* CPS was poorly immunogenic in mice and rabbits [18]. However, use of CPS-protein conjugates did elicit improved immune responses such that the resulting antisera could be used to probe structural diversity of CPS. In agglutination assays, antibodies raised against CPS from strain C7184 (Table 1), for example, cross-reacted with only three other strains out of 32 tested [18], while antisera raised against strain MO6-24/O CPS cross-reacted with four out of 21 clinical isolates and none of 67 environmental strains examined [19]. In the latter study, NMR and HPAEC analyses of purified CPS from a subset of strains revealed most had distinct sugar composition and structure [19]. Such preliminary studies led to the development of a chemotyping method for *V. vulnificus* CPS, which involved HPAEC analysis coupled with electrochemical detection and computer automation. Analysis of CPS from 120 *V. vulnificus* clinical and environmental isolates revealed that they could be divided into 94 ‘carbotypes’, each differing in their carbohydrate composition [20]. No obvious correlation between carbotype and pathogenic potential of isolates was evident, though the possibility that some common feature (i.e., epitope) among clinical strains may exist could not be excluded [20]. 

## 4. Further Insights Regarding CPS Functions That Contribute to Virulence 

As described earlier, studies conducted in the early to mid-1980s firmly established a correlation between virulence and CPS production in *V. vulnificus*. Moreover, specific virulence-related functions, such as antiphagocytic ability and resistance to the bacteriocidal action of human serum, appeared to be associated with CPS. Kreger et al. 1981 reported that a virulent strain of *V. vulnificus* was more resistant to phagocytosis by human polymorphonuclear (PMN) leukocytes than an attenuated strain, and their evidence suggested the possible involvement of an antiphagocytic antigen on the surface of the virulent isolate, which was later identified as an acidic polysaccharide [6,7]. Similarly, following exposure to opsonins present in NHS, highly virulent strains showed reduced uptake by human PMNs compared to less virulent isolates [21]. It was later shown that *V. vulnificus* also resisted phagocytosis by murine peritoneal macrophages in the absence of serum opsonins, a result which reinforced the notion that the surface of virulent cells (i.e., a molecule on the surface) had antiphagocytic properties [22]. 

Highly virulent *V. vulnificus* isolates also displayed resistance to the bacteriocidal action of NHS [21,23]. In assays designed to measure complement activation for killing, *V. vulnificus* activated significantly less complement than other pathogenic *Vibrio* spp. [23]. Multiple studies have since shown that opaque-colony-type variants of *V. vulnificus* are resistant to killing by NHS, while isogenic translucent variants are susceptible, which supports the hypothesis that CPS protects against serum killing [9,24]. It is possible that the negative charge of CPS confers resistance to the antibacterial components present in NHS [9,24]. Moreover, CPS may conceal immunogenic features on the surface of *V. vulnificus* cells that would otherwise facilitate the innate immune responses of the host, including phagocytosis by PMNs and macrophages and complement-mediated killing [2,3].

Septic shock typically results from excessive and systematic induction of inflammatory cytokines. For septicemia caused by *V. vulnificus*, such cytokines include tumor necrosis factor alpha (TNF-α), interleukin 1 beta (IL-1β), IL-6, and IL-8 [2,3]. A number of pathogen-associated molecules, including surface molecules, can interact with toll-like receptors (TLRs) present on host immune and non-immune cells to activate cytokine gene expression in a NF-κB-dependent manner during infections [25]. Given the evidence that CPS is a major surface molecule on virulent *V. vulnificus* cells, a potential role for it in host cytokine activation was investigated. When exposed to either an encapsulated strain of *V. vulnificus* or purified CPS from that strain, the production of TNF-α was stimulated in vitro in human peripheral blood mononuclear cells or in vivo in mice [26]. Lee et al. (2010) then examined the effects on cytokine induction and related functions for human intestinal epithelial cells exposed to an encapsulated *V. vulnificus* clinical isolate versus an isogenic CPS mutant. The encapsulated strain induced significantly greater IL-8 production and NF-κB transcriptional activity than the mutant, and TLR-2 mRNA and protein levels were also greater following exposure to the encapsulated strain. Moreover, IL-8 production and NF-κB activity were also significantly induced following exposure to purified CPS, effects which could be blocked by the addition of anti-TLR2 antibodies. Thus, *V. vulnificus* CPS appears to induce inflammatory cytokine production via a TLR2/NF-κB-dependent route [27]. 

An additional potential virulence-related function of the CPS of *V. vulnificus* is its apparent effect on production and spatial distribution of outer membrane vesicles (OMVs) [28]. As with other Gram-negative pathogens, OMVs may contribute to pathogenicity of *V. vulnificus*, a hypothesis supported by the finding that OMVs of this species induced death of epithelial cells by delivery of the cytolysin-hemolysin VvhA [29].

## 5. CPS Genetics and Biosynthesis 

CPS biosynthesis and genetics in *Escherichia coli* has served as the model for characterization of capsules from other bacterial species. Based on CPS composition, as well as the underlying arrangement and complement of biosynthetic and transport genes, capsules of *E. coli* have been divided into four groups [30]. To date, the groups relevant for characterization of CPS production in *V. vulnificus* have been Groups 1 and 4. Capsules within Groups 1 and 4 are also interesting in that they are related to LPS O antigens [30]. 

Group 1 capsules of *E. coli* contain acidic sugar residues (e.g., uronic acids) as part of their repeat-unit structures, while Group 4 capsules are notable for the presence of acetomido sugars. For both capsular groups, individual repeat units are assembled on the lipid carrier undecaprenyl phosphate (und-P) with assembly occurring on the inner side of the inner membrane. Glycosyltransferase enzymes add the particular sugar residues that compose the repeat unit to the lipid carrier. In the case of Group 1 capsules, the WbaP protein, which is a member of the polyisoprenyl-phosphate hexose-1-phosphate transferase protein family, is the initiating glycosyltransferase, and it catalyzes the transfer of galactose-1-phosphate or glucose-1-phosphate to und-P, while WecA (polyisoprenyl-phosphate N-acetylhexosamine-1-phosphate transferase family) performs the initial transfer of glucosamine-1-phosphate to und-P during Group 4 CPS synthesis. Following assembly, the completed und-P-linked repeat units for Group 1 and 4 capsules are translocated across the inner membrane by the Wzx flippase (polysaccharide transport (PST)-1 family; E.C. 7.5.2.6). The individual repeat units are then joined together on the periplasmic side of the inner membrane by the Wxy polymerase (no specified protein family), which transfers the growing polysaccharide from its lipid carrier to the newly translocated repeat unit [30]. 

CPS undergoes further polymerization and is relocated across the outer membrane of *E. coli* in a process that involves the Wza, Wzb, and Wzc proteins. Wza (outer membrane auxiliary (OMA) family) forms a multimeric pore through which the CPS traverses the outer membrane. Wzc is a tyrosine autokinase (and a member of the membrane periplasmic auxiliary (MPA)-1 protein family), which is anchored in the inner membrane but extends into the periplasm, and it is required for higher-level polymerization of CPS. Wzb (protein tyrosine phosphatase (PTP) family; E.C. 3.1.3.48) is the cognate phosphatase for Wzc. Both phosphorylation of Wzc and its dephosphorylation by Wzb are important for CPS synthesis [31]. Although Wza, Wzb, and Wzc proteins are required for both Group 1 and 4 CPS synthesis, a distinguishing feature is that the *wza*, *wzb*, and *wzc* genes are part of the Group 1 CPS biosynthetic and transport locus, which also includes *wzx* and *wzy*, while in Group 4 strains, the *wza*, *wzb*, and *wzc* genes are encoded at a separate location from the main locus [30]. 

Sequencing of the genetic region flanking the sites of transposon insertions in acapsular mutants of various strains provided insights into the genetics of CPS synthesis in *V. vulnificus*. For strain MO6-24/O, the presence of both biosynthetic and transport genes, including *wza*, *wzb*, and *wzc*, at a single locus and in an apparent single long operon configuration, coupled with the acidic composition of the MO6-24/O capsule, allowed for designation of a Group 1 capsule and CPS operon for this strain [15,32,33]. 

Meanwhile, a partial genetic organization of the CPS locus in strain 1003(O) (Table 1) was reported [12,14]. Although the presence of a *wecA* homolog was suggestive of a Group 4 designation, a final group determination for strain 1003(O) was not possible from the available data [12]. 

Similar analysis of strain ATCC 27562 led to the identification of its CPS locus which had characteristics of Group 1 organization (i.e., linkage of *wza*, *wzb*, and *wzc* with CPS biosynthetic genes); however, the presence of a *wecA* glycosyltransferase gene at this locus, coupled with the CPS composition for this strain, supported a Group 4 designation [17,34,35]. 

The findings of linkage between CPS transport and biosynthetic genes even for the Group 4 strain ATCC 27562 raise the possibility that such gene organization may be common in this species. As an initial test of this notion, we determined the position of the *wza*, *wzb*, and *wzc* genes relative to the CPS biosynthetic locus in 21 *V. vulnificus* strains whose complete genome sequences were available in the NCBI database. We found evidence for such linkage in 14 of those strains, while five strains showed no linkage between transport and biosynthetic genes, and two could not be determined due to limited sequence identity of the genes involved. Among the 14 strains that showed linkage, the majority possessed a *wbpA* homolog, though there were also multiple strains, besides ATCC 27562, that contained *wecA* (Mukerji and Pettis, unpublished results).

Interestingly, the CPS locus of strain ATCC 27562 was also found to be largely conserved and syntenous with one in the marine bacterium *Shewanella putrefaciens* strain 200 [34]. The average GC content for both loci was lower than that of either host genome, and a number of the encoded proteins showed highest similarity to proteins of Gram-positive and archaeal origins. These findings led to the authors’ proposed hypothesis that these CPS loci were acquired by their *V. vulnificus* and *S. putrefaciens* host strains via horizontal gene transfer (HGT) events from an unrelated microbe [34]. 

In a follow-up study, the potential for HGT acquisition of CPS loci by *V. vulnificus* was explored further. It was found that *V. vulnificus* strains induced to a state of competence during transformation following exposure to chitin were able to incorporate into their genome exogenous DNA, including CPS locus sequences tagged with a selectable marker. In some cases, full or partial CPS locus sequences were incorporated, which resulted in a change in CPS carbotype [36]. It is possible that the notable CPS carbotype diversity in *V. vulnificus* [20] has been the result, at least in part, of HGT acquisition of CPS loci, which can result in new combinations of biosynthetic genes and therefore new carbotypes of CPS [36].

## 6. CPS Phase Variation 

Both opaque and translucent colony types of *V. vulnificus* strains were often readily observed [8,9,37], thus raising the possibility that phase variation between these two morphotypes was occurring. Rates of switching from opaque to translucent colonial variants ranged from 10^−4^ to 10^−5^ [9,11,38]; in some cases, phenotypic switching from translucent back to opaque at rates on the order of 10^−4^ was also observed [9,11]. Such frequencies, which were orders of magnitude greater than expected if spontaneous mutations were responsible, supported the existence of a reversible CPS phase variation mechanism(s).

Chatzidaki-Livanis et al. (2006) then studied the process of phase variation in more detail at both the phenotypic and genotypic levels for *V. vulnificus* strains that contained a Group 1 CPS operon. They found evidence for two distinct types of translucent isolates derived from an opaque parent. One translucent type retained the ability to switch back to the opaque form, while the other type showed no evidence of switching and thus appeared to be phase locked. In electron micrographs of negatively stained cells, translucent isolates capable of phase switching showed reduced amounts of CPS on the cell surface as compared to the opaque parent, while phase-locked translucent isolates appeared to be acapsular. At the genetic level, PCR analysis revealed that phase-locked translucent isolates had typically undergone deletion of the *wzb* gene along with some flanking sequences within the Group 1 CPS operon (e.g., TR2 isolates). Meanwhile, translucent isolates that retained the ability for reversible phase variation (TR1) possessed no mutations at all within their Group 1 operons [32]. 

**Figure 2:**
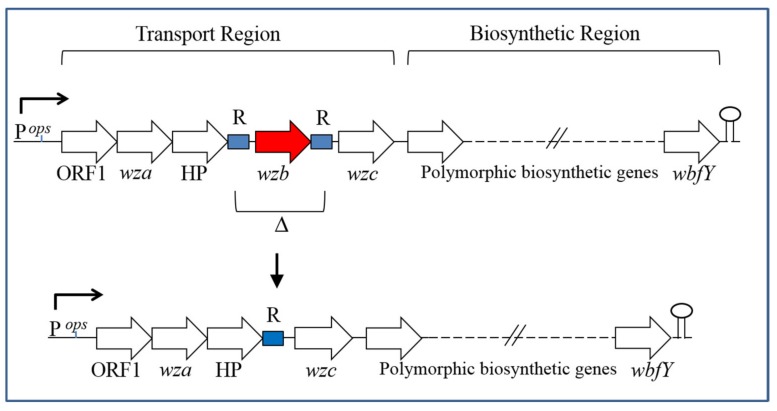
Model for generation of phase-locked translucent variants based on Chatzidaki-Livanis et al. (2006). The upper portion of the figure depicts the general organization of the Group 1 CPS operon in *V. vulnificus*, including the positions of the essential *wzb* phosphatase gene and repeat sequence (R) regions flanking that gene. For strains containing allele 1 of this operon, each R region contains multiple copies of the octamer (ACAGGACC). Allele 2 strains have an additional R region (not shown) located between *wza* and HP (hypothetical protein gene), and each R region contains multiple copies of (A/CCTAGG/AAA/C) [32]. Starting with an opaque variant, homologous recombination between R regions with deletion of intervening sequences, including *wzb*, would generate a phase-locked translucent variant (i.e., as depicted in the lower portion of the figure) [32]. The relative positions of the operon promoter (P), *ops* element, and Rho-independent transcription terminator hairpin structure are indicated.

In the same study, examination of Group 1 operon sequences revealed a probable mechanism for the creation of phase-locked translucent variants and uncovered allelic differences at this locus. Opaque strains containing allele 1 (e.g., MO6-24/O) were distinguished by having multiple copies of the same octamer sequence (ACAGGACC) repeated directly and sequentially both upstream and downstream of *wzb*. Opaque strains containing allele 2 also had multiple direct repeats present within their Group 1 transport regions, including on either side of *wzb*. However, the sequence of these repeats was more variable (A/CCTAGG/AAA/C) and copies were interspersed with other unrelated sequences. As shown in Figure 2, a potential mechanism for the generation of phase-locked translucent variants would then consist of homologous recombination involving repeat sequences flanking *wzb*, which would result in deletion of the intervening sequence [32]. 

The intermediate (Int) variant reported for certain strains of *V. vulnificus* appears to be analogous to the TR1 isolate, in that both contained an intact *wzb* gene and could switch back to opaque [3,32,39]. RT-PCR analysis suggested the possibility of transient down-regulation of *wzb* expression in newly formed Int colonies [39]. The potential for expression differences in Group 1 CPS operon genes in opaque versus TR1 (Int) derivatives remains an interesting possibility to be explored. 

A number of environmental factors affecting CPS phase variation in *V. vulnificus* have now been identified. Significantly greater switching from opaque to translucent was seen during static growth as compared to growth under aeration for most strains analyzed [40]. Greater opaque-to-translucent switching was also seen at elevated (e.g., 37 °C) versus lower (23 °C) temperatures [40]. These authors also compared phase switching from opaque to translucent in response to temperature for C type (i.e., predominantly clinical isolates) versus E type (predominantly environmental isolates) of *V. vulnificus* strains, which had previously been subdivided into those types by using a randomly amplified polymorphic DNA-PCR method [41]. Interestingly, C-type strains were significantly less prone than E type strains to switch to the translucent form at all temperatures examined including those relevant to human infection [40]. 

In an interesting twist on the phase variation phenomenon, it was found that opaque *V. vulnificus* strains appeared translucent when grown under anaerobic conditions [42]. However, such strains regained opacity when exposed to an aerobic environment. In line with these phenotypic results, it was found that expression of the *wza*, *wzb*, and *wzc* genes were all significantly downregulated under anaerobic conditions. These results raise the possibility that CPS production may be downregulated during earlier anaerobic stages of human infection, such as colonization of the intestinal tract [42]. 

Calcium (Ca^2+^) was found to significantly enhance colonial phase variation of *V. vulnificus*. Some opaque strains switched predominantly to translucent in the presence of elevated calcium while other strains switched mostly to a biofilm-proficient rugose form [43]. Increased manganese (Mn^2+^) concentrations also significantly enhanced switching of opaque strains to translucent or rugose phenotypes [44]. PCR analysis of translucent isolates obtained following growth in elevated Ca^2+^ or Mn^2+^ revealed the presence of both *wzb*+ translucent variants, as well as many that had undergone deletions of *wzb*. Thus, elevated Ca^2+^ or Mn^2+^ concentrations, which the bacterium may encounter in its natural estuarine environment, likely up-regulate existing phase variation mechanisms [43,44]. 

In its natural marine habitat, *V. vulnificus* becomes concentrated in filter feeders such as oysters. Using an oyster model of infection that was reduced for indigenous *V. vulnificus* populations, Srivastava et al. (2009) studied the ability of various *V. vulnificus* mutants and phase variants to colonize and persist in oysters. Reminiscent to what was seen previously in mice [11], it was found that following infection of oysters with a T1 (TR1) translucent isolate, the strain was subsequently recovered in the opaque form at a substantial frequency (i.e., 72% of the colony-forming units recovered from the oysters were opaque while only 28% remained translucent) [45]. The predominance of the opaque form over the translucent one in oysters was supported by co-infection experiments [45], as well as by previous observations that translucent *V. vulnificus* isolates appear to be more susceptible than opaque to phagocytosis by oyster hemocytes [46].

Some regulatory systems also appear to affect phase variation in *V. vulnificus*. GacS/GacA is a two-component signal transduction system that regulates a variety of functions in Gram-negative bacteria, including virulence factor production [47]. While a *gacA* knockout strain of *V. vulnificus* retained colony opacity, its ability to undergo phase switching to translucent was significantly reduced [48]. Among the transcripts significantly down-regulated in the *gacA* mutant was *rpoS* [48], which encodes the sigma factor (σ^S^) that is critical for stationary phase gene expression. Phase variation in *V. vulnificus* has been purported to be a response to stationary growth [32]; thus, the results for *gacA* and *rpoS* may shed light on one regulatory mechanism controlling the appearance of new phase variants in this species. 

In a separate study, insertion into or deletion of the *rseB* gene, produced mutants that appeared less opaque than the parental strain and, upon passaging, yielded mixed populations of opaque and translucent colonies at a high frequency [49]. The RseB protein is a periplasmic negative regulator of σ^E^, which controls expression of genes critical for maintaining cell envelope integrity during stress (i.e., the extracytoplasmic stress response) [50]. A strain overexpressing σ^E^ did not recapitulate the colonial phenotype and phase-switching characteristics of *rseB* mutants [49]. Nevertheless, the results suggested a potential role for *rseB* in regulating phase variation in *V. vulnificus*, although the extent to which CPS expression specifically was affected in these mutants was not determined [49].

## 7. Other Regulation of CPS 

While not contributing to phase variation per se, other environmental and genetics factors have also been identified that affect CPS expression in *V. vulnificus*. Using a fluorescence-based flow cytometry method specific for CPS, Wright et al. (1999) determined that encapsulated strains showed maximal CPS expression during logarithmic growth as opposed to the stationary phase. Moreover, opaque cells produced significantly more CPS when grown at 30 °C rather than 37 °C [51]. 

Biofilm production in *V. vulnificus* involves an exopolysaccharide that is distinct from CPS and is encoded by its own exopolysaccharide gene cluster [52,53]. Joseph and Wright (2004) compared the ability of isogenic opaque and translucent variants to form biofilms and found that translucent isolates produced significantly more biofilm than opaque. The authors concluded that CPS inhibits biofilm formation in *V. vulnificus* [54]. Lee et al. (2013) then showed that exogenous addition of purified CPS to cultures of a translucent variant greatly reduced biofilm formation, while addition of a CPS-degrading enzyme to the parental opaque strain significantly increased its ability to form biofilms. They next demonstrated that CPS inhibits biofilm formation in a quorum sensing-dependent manner. A mutant defective for the quorum sensing master regulator SmcR had a translucent phenotype on agar plates, and transcription of the Group 1 CPS operon was reduced tenfold in the *smcR* mutant compared to the opaque wild type parent. A SmcR binding sequence in the promoter region for this operon was demonstrated by using DNaseI footprinting assays involving recombinant SmcR protein. Overall, the authors suggested that CPS may play an important role in regulating the size of mature biofilms by limiting their continual growth [55]. 

In a follow-up study, CPS production and CPS Group 1 operon gene expression were found to be significantly reduced in cells subjected to heat shock at 42 °C. Basal level transcription of the CPS operon under these conditions was shown to be the result of enhanced degradation of SmcR by proteases ClpPA and Lon [56]. 

Lastly, the production of a variety of surface molecules in Gram-negative species has been shown to be regulated by the antiterminator RfaH protein, which functions to prevent premature transcriptional termination at Rho-dependent sites within the cognate operons for these molecules. RfaH is recruited to the transcription elongation complex by binding a small conserved operon polarity sequence (*ops*), which is typically located upstream of the first gene within operons [57,58]. Given that an *ops* element was previously described [33] within the Group 1 CPS operon of *V. vulnificus* (Figure 2), an effort was undertaken to evaluate the role of RfaH in CPS production in this species. It was found that knockout of the *rfaH* gene in an opaque parental strain produced a translucent derivative that appeared phase locked. Moreover, distal, but not proximal, gene expression within the CPS Group 1 operon was significantly reduced in the *rfaH* mutant, and it was highly sensitive to killing by NHS. Thus, CPS production and serum survival in *V. vulnificus* were found to be dependent on RfaH antitermination control [59].

## 8. Concluding Remarks 

**Figure 3:**
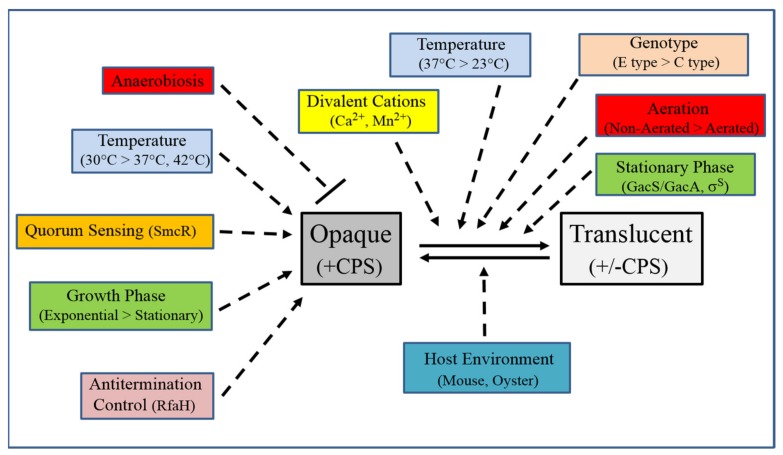
Summary of known environmental, genetic, and regulatory factors that affect CPS production in *V. vulnificus*. CPS production in opaque variants is negatively affected (depicted by dashed line with bar) by anaerobiosis [42], but positively affected (dashed arrows) by lower growth temperature [51,56], quorum sensing as mediated by SmcR [55], exponential growth [51], and antitermination control via RfaH [59]. Phase switching from opaque to translucent (solid arrow) is positively affected by divalent cations Ca^2+^ and Mn^2+^ [43,44], higher growth temperature [40], E-type genotypes [40], non-aerated growth [40], and stationary phase growth, with the latter effect potentially being mediated by GacS/GacA and σ^S^ [48]. Host environments such as mouse [11] and oyster [45] promote phase variation from translucent to opaque, but to what extent, if any, the rate of phase switching is affected by these environments remains to be determined. See text for more details regarding these various factors.

The CPS of *V. vulnificus* plays pivotal roles in circumventing the innate immune response during infection of the human host, and it appears to also stimulate a portion of the inflammatory cytokine response to this pathogen. Substantial CPS carbotype diversity among *V. vulnificus* strains is underscored by considerable genetic diversity, which may be the result of HGT events that lead to new combinations of CPS biosynthetic genes. As summarized in Figure 3, CPS production is controlled by numerous environmental, genetic, and regulatory factors, including those that affect phase variation between virulent encapsulated opaque variants and attenuated translucent variants, which possess little or no CPS on their cell surface. Further study of CPS production and its regulation in *V. vulnificus* should continue to provide important insights that may ultimately lead to improved disease management of this deadly pathogen.

## Figures and Tables

**Table 1:** *V. vulnificus* strains discussed in this review.

Strain	Source	CPS Phenotype	CPS Group
ATCC27562^1^	Clinical	Opaque	4
FCC	Clinical	Opaque^2^	N.D.^3^
MO6-24/O	Clinical	Opaque	1
MO6-24/T	Phase variant of MO6-24/O	Translucent	1
BO62316	Clinical	Opaque	N.D.
C7184	Clinical	Opaque	N.D.
1003(O)	Clinical	Opaque	N.D.

^1^*V. vulnificus* type strain. ^2^ Phenotype based on capsular polysaccharide (CPS) staining results in Amako et al. (1984). ^3^ Not determined.

## Abbreviations

CPS	Capsular polysaccharide
LPS	Lipopolysaccharide
NHS	Normal human serum
NMR	Nuclear magnetic resonance
HPAEC	High performance anion-exchange chromatography
TNF-α	Tumor necrosis factor alpha
IL	Interleukin
Und-P	Undecaprenyl phosphate
